# Serum Polychlorinated Biphenyls Increase and Oxidative Stress Decreases with a Protein-Pacing Caloric Restriction Diet in Obese Men and Women

**DOI:** 10.3390/ijerph14010059

**Published:** 2017-01-10

**Authors:** Feng He, Li Zuo, Emery Ward, Paul J. Arciero

**Affiliations:** 1 Human Nutrition and Metabolism Laboratory, Health and Exercise Sciences Department, Skidmore College, 815 North Broadway, Saratoga Springs, NY 12866, USA; fhe@csuchico.edu (F.H.); emeryward@gmail.com (E.W.); 2 Department of Kinesiology, California State University-Chico, Chico, CA 95929, USA; 3 Radiologic Sciences and Respiratory Therapy Division, School of Health and Rehabilitation Sciences, The Ohio State University College of Medicine, The Ohio State University Wexner Medical Center, Columbus, OH 43210, USA; zuo.4@osu.edu

**Keywords:** polychlorinated biphenyls (PCBs), oxidative stress, caloric restriction, intermittent-fasting

## Abstract

The purposes were to compare the effects of a: (1) 12-week P-CR weight loss (WL) diet (Phase 1) between obese men and women and; (2) 52-week modified P-CR (mP-CR) vs. heart healthy (HH) weight maintenance (WM) diet (Phase 2) on serum PCBs and oxidative stress biomarkers (thiobarbituric acid reactive substances, TBARS; total antioxidant capacity, TAC) in 40 obese participants (men, *n* = 21; women, *n* = 19). Participants received dietary counseling and monitoring of compliance. PCBs, TBARS, and TAC were assessed at weeks −1 (CON), 12 (WL), and 64 (WM). Following WL (Week 12), concomitant with reductions in TBARS (0.24 ± 0.15 vs. 0.18 ± 0.11 µM; *p* < 0.01), PCB serum concentrations (86.7 ± 45.6 vs. 115.6 ± 65.9 ng/g lipid; *p* < 0.01) and TAC (18.9 ± 2.6 vs. 19.9 ± 2.3 nmol/mL; *p* < 0.02) were increased similarly in men and women. At the end of WM (Week 64), a significant effect of time × group interaction was observed for % change in PCB 170 and 187; whereby mP-CR values were higher compared to HH (PCB170: 19.31% ± 26.48% vs. −6.61% ± 28.88%, *p* = 0.02; PCB187: −3.04% ± 17.78% vs. −21.4% ± 27.31%, *p* = 0.04). PCB changes were positively correlated with TBARS levels (*r* > 0.42, *p* < 0.05) and negatively correlated with body weight, fat mass, and abdominal fat (*r* < −0.46, *p* < 0.02). Our results support mobilization of stored PCBs as well as enhanced redox status following a 12-week P-CR WL diet. Additionally, a 52-week mP-CR WM diet demonstrated an advantage in preventing weight gain relapse accompanied by an increase in circulating PCBs compared to a traditional HH diet.

## 1. Introduction

Dietary weight loss (WL) interventions lower cardiometabolic disease risk factors, including visceral/abdominal fat, adipokines, oxidative stress, and inflammatory markers [1,2,3,4]. Protein-pacing caloric restriction (P-CR) WL remains one of the most efficacious interventions to treat obesity and its associated conditions in both short- and long-term randomized control trials compared with other diets [5,6,7,8]. We have recently shown protein-pacing (P; 4–6 meals/day @ ≥25% of total kcals/day) and caloric restriction (CR; ≤1500 kcals/day), including intermittent-fasting (IF, 1 day per week of <500 kcals/day) WL interventions to successfully promote WL and enhance body composition and cardiovascular health [6,9].

Interestingly, although women generally have greater overall adiposity, men tend to have more central/abdominal obesity that is more metabolically active and therefore may release more polychlorinated biphenyls (PCBs) into the circulation than woman [10]. Indeed, fat mass loss is generally regarded as beneficial during weight loss (WL) interventions [11], but it is also associated with an increased release of PCBs from fat depots [12]. To our knowledge, there is a paucity of studies directly comparing the sex difference in response to P-CR WL induced plasma increase in PCBs.

Although it is likely to be the first step in the detoxification process, a WL-induced rise in plasma toxins (such as PCBs) and organohalogenated contaminants has been observed [13,14]. PCBs are common environmental organic pollutants, and may be encountered through contaminated environmental sources such as soil and water, yet such hazards have been greatly reduced after the banning of the use of industrial PCBs in 1979 [15,16]. Humans are exposed to PCBs mainly through food consumption, which tend to accumulate in fatty acids of the food sources [15,16] and eventually stored in adipose tissue, exerting adverse effects on human health via metabolic and endocrine disruption [17,18,19]. Previous data shows WL-induced rise in plasma pollutants is related to in vitro subcutaneous adipocyte basal lipolysis and reduced skeletal muscle oxidative capacity, which may be risk factors for weight gain [20]. Several recent investigations have examined the relationship between weight change and oxidative stress, especially in the context of increased serum toxin levels during WL [11,13], however the influence of diet quality and sex differences has not been investigated.

Thus, it is highly desirable to identify effective dietary strategies that minimize oxidative stress during WL induced increase in serum PCBs. From an overall health perspective, an increased mobilization of stored PCBs into the serum, concomitant with enhanced redox status, is preferred during WL. However, no study has investigated sex differences in response to P-CR WL-induced changes in circulating organic pollutants (PCBs) and oxidative stress response in obese adults. Thus, a primary aim of this study was to compare changes in serum PCBs and oxidative stress biomarkers (TBARS, TAC) between obese men and women following a short-term P-CR diet intervention (Phase 1, WL; weeks 1–12).

While it is well-accepted that most diet interventions can effectively induce WL in the short-term (<3–6 months), there is a paucity of evidence investigating the long-term (≥52 weeks) effects of a modified P-CR (mP-CR, 1–2 days per month of IF) diet during weight maintenance (WM) on serum PCBs and oxidative stress in obese adults following short-term WL. Therefore, the other major aim of the current study was to compare the long-term (52 weeks) efficacy of an mP-CR versus the traditionally prescribed heart healthy (HH) diet following an initial 12 weeks WL period (Phase 1, weeks 1–12) on plasma PCBs and oxidative stress biomarkers in obese men and women (Phase 2, WM; weeks 13–64). We hypothesized that: (1) a P-CR diet would be equally effective at promoting WL, mobilizing stored PCBs, and reducing oxidative stress in obese men and women (Phase 1, WL; weeks 1–12) and; (2) during long-term (52 weeks) WM (Phase 2; weeks 13–64), an mP-CR diet would sustain PCB release while maintaining reduced oxidative stress compared to a HH diet.

## 2. Materials and Methods

### 2.1. Participants

**Figure 1:**
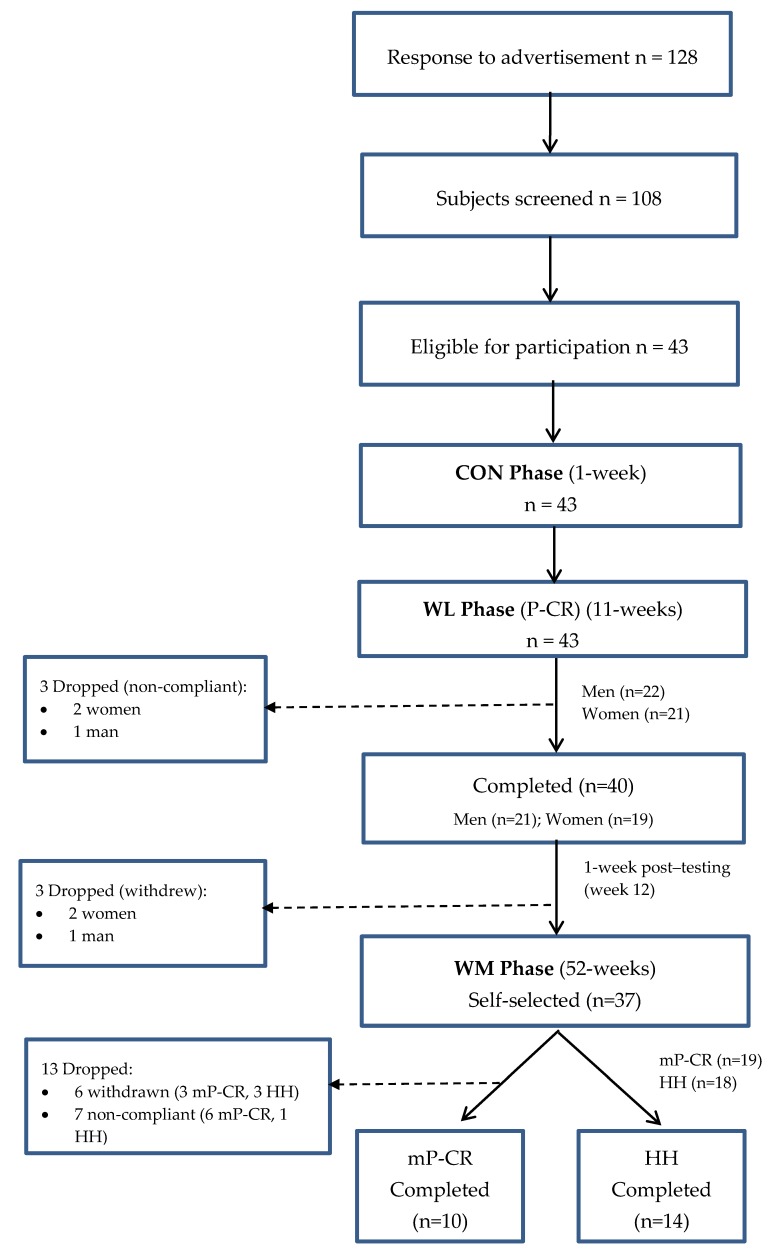
CONSORT of Participant Flow Chart.

Physical characteristics of the participants, changes in body composition, energy expenditure, biomarkers, and cardiovascular outcomes in response to a P-CR WL diet and comparison of long-term mP-CR versus HH diet have been reported previously [6,9], and only the necessary minimum will be repeated in this paper. All experiments were carried out in 1 series; the original study design aimed to compare body composition, biomarkers, cardiovascular, toxin, and oxidative stress responses between: (1) obese men and women following a short-term P-CR WL diet and (2) mP-CR and HH diet following long-term WM. A total of 128 individuals from the Saratoga Springs, NY area, responded through emails, flyers and local newspapers to advertisements regarding the study. A total of 108 subjects were initially screened, of which 43 were eligible for participation (Figure 1).

Each participant provided informed written consent in adherence with the Skidmore College Human Subjects review board prior to participation, and the study was approved by the Human Subjects Institutional Review Board of Skidmore College (IRB #: 1307-347). All experimental procedures were performed in accordance with the Federal Wide Assurance and related New York State regulations, which are consistent with the National Commission for the Protection of Human Subjects of Biomedical and Behavioral Research and in agreement with the Helsinki Declaration as revised in 1983. Isagenix International staff were provided interim data summary reports throughout the study but did not have access to any of the raw data. This study was registered with ClinicalTrials.gov Identifier: NCT02525419.

### 2.2. Experimental Design

#### Study Timeline

**Figure 2:**
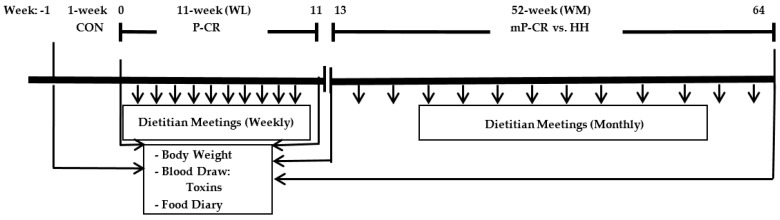
Study Timeline.

This 64 week nutritional intervention consisted of two consecutive dietary intervention phases: (a) 12-week P-CR WL diet (1-week baseline control, CON; 11-week WL) comparing men and women (Phase 1, WL; weeks 1–12) and (b) a 52 weeks comparison of mP-CR versus a heart healthy (HH) diet (Phase 2, WM; weeks 13–64). Immediately following the initial WL phase (12-week), participants self-selected to either mP-CR or approved “heart healthy” diet (HH) and maintained their diet plan for another 52 weeks (1 year) (Figure 2).

All laboratory testing procedures (see below) were completed following baseline week-1 (CON), week-12 (WL), and week-64 (WM). Please note, the current manuscript reports data on correlation coefficients for body weight, total and abdominal fat mass with PCBs and oxidative stress biomarkers only. Absolute body composition changes are reported in a separate manuscript [6]. Participants were asked to maintain habitual eating patterns and record their dietary food logs for two days during CON in order to maintain stable weight. Similarly, during WL (P-CR) diet intervention, participants were asked to maintain current level of physical activity (sedentary—low activity) and to refrain from adopting new exercise regimens. At weeks 0, 12 and 64, all participants arrived for testing between the hours of 6:00 a.m.–9:00 a.m., fasted overnight, and were measured for body weight and waist circumference. Participants were then asked to rest for 15-min in a supine position in a quiet and dimly lit room before a 30-min resting metabolic rate (RMR) measurement followed by a fasted blood draw (~20 mL) for serum PCBs and oxidative stress measures (see Laboratory Testing Procedures below). Following CON baseline testing, participants were given detailed instructions on their WL dietary guidelines (see Dietary Intervention) and scheduled their weekly dietitian meeting. At the beginning of the WM (Phase 2), participants in both groups (mP-CR and HH) met with a licensed registered dietitian with more than 10 years dietary counseling experience and continued to do so on a monthly basis.

## 3. Dietary Intervention

### 3.1. Weight Loss (WL) Phase (Weeks 1–12): P-CR Diet

**Figure 3:**
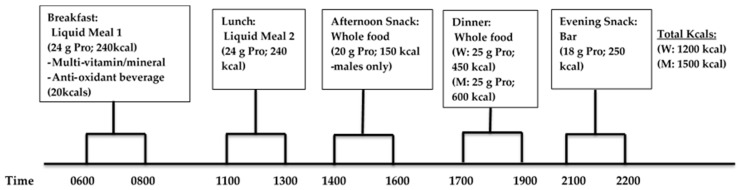
Daily timing and meal content during Phase 1 (Weight Loss, WL; P-CR).

Participants consumed a P-CR diet for 6 days of the week, and incorporated an intermittent-fasting diet (330–430 kcals/day) on the remaining day of the week in conjunction with weekly dietary counseling by a registered dietitian. The timing and frequency of meals and protein consumption were novel and essential components to the study. Each meal eaten during WL consisted of approximately 20–25 g servings of high-quality protein in either supplement or whole food form. Subjects were instructed to eat ~4–5 meals per day and consume their breakfast liquid meal replacement within one hour upon waking in the morning and eat approximately every 3 h during the day consuming their final evening snack within 1 h of going to bed at night. During WL (and WM), the following timing of meals was recommended (Figure 3): breakfast between 6:00 a.m. and 8:00 a.m., lunch 11:00 p.m.–1:00 p.m., afternoon snack 2:00 p.m.–4:00 p.m., dinner 5:00 p.m.–7:00 p.m., and evening snack bar 9:00 p.m. and 10:00 p.m.

Liquid protein supplements were distributed to each participant in powder form and mixed with water for each daily feeding (breakfast and lunch; kcals: 480 kcals/day total) and the protein bar was consumed with water. The daily total macronutrient intake consisting of both liquid shakes (breakfast and dinner) and bar meal (evening snack) replacements in combination with whole food choices (afternoon snack men only, dinner) was 1200 and 1500 kcals for women and men, respectively. In these plans, men and women consumed a fixed amount of calories since they were assigned with the same fixed total amount of calories in each meal based on the estimated total energy expenditure (Table 1). The macronutrient distribution of meals (30% PRO, 45% CHO, and 25% FAT) has been used successfully in our lab to induce an energy deficit without compromising lean body mass [4].

On intermittent-fasting days, daily energy intake consisted of 330 and 430 kcals per day for women and men, respectively (Table 2).

### 3.2. Weight Loss Maintenance (WM) Phase (Weeks 13–64); Modified P-CR (mP-CR) or Heart Healthy (HH) Dietary Interventions

Beginning at week 13, participants self-selected into an mP-CR or a HH diet intervention in combination with monthly dietary counseling with a registered dietitian. By design, subjects were instructed to adhere to the respective guidelines of each diet with no restriction on total food intake or physical activity to more closely resemble a “free-living” pattern of energy balance. Participants in the mP-CR group followed a diet similar to the WL phase but were only provided 2 meal replacements per day (either 2 protein powder packets or 1 protein powder packet and 1 meal replacement bar) and the remaining 2–3 meals were whole foods. In addition, mP-CR subjects performed intermittent-fasting 1–2 times per month.

Participants in the HH group followed the dietary guidelines that are in compliance with the National Cholesterol Education Program Therapeutic Lifestyle Changes (TLC) diet (i.e., <35% of kcal as fat; 50%–60% of kcal as carbohydrates; <200 mg/day of dietary cholesterol; and 20–30 g/day of fiber). Food was not provided to the subjects for any of the meals. Instead, all subjects (HH and mP-CR) met with a Registered Dietician monthly to learn how to make healthy eating choices that are in compliance with their respective meal plans. Participants also had access to additional counseling with the registered dietitian if necessary.

### 3.3. Compliance

The specific details of compliance have been previously published [6]. Briefly, all participants met with a Registered Dietitian weekly during WL and monthly during WM to incorporate healthy eating strategies and nutritional counseling while successfully consuming their respective total macronutrient caloric amounts and dietary meal plans.

Throughout the intervention, 2-day food records were used to verify compliance to the diets (P-CR, mP-CR, HH) using The Food Processor SQL Edition (version 10.2.0 ESHA Research, Sale, OR, USA, 2012), and were analyzed by a single trained operator to alleviate inter-investigator variation (EW). In addition, participants were provided a checklist to monitor their adherence to the intermittent-fasting day regimen.

## 4. Laboratory Testing Procedures

### 4.1. Body Weight, Height, Body Mass Index (BMI), and Composition Assessment

Please note, only correlation coefficients for body weight, total and abdominal fat mass with serum toxins and oxidative stress biomarkers are reported in the current manuscript. Absolute changes for all body composition variables are presented in a separate manuscript [6]. Body weight and height measurements were recorded during each testing visit in light minimal clothing and without shoes using an electronic scale, and used to calculate body mass index (BMI; weight in kilograms/(height in meter)^2^. Total fat mass (FM) and abdominal fat mass was determined by dual energy X-ray absorptiometry (iDXA; software version 13.6, model iDXA; GE Healthcare, Madison, WI, USA) at weeks 0, 11, and 64 (Figure 2).

### 4.2. Oxidative Stress Measurements (TBARS and TAC)

Lipid peroxidation was analyzed with TBARS assay (R&D Systems, Minneapolis, MN, USA) [21,22]. This required 300 µL of plasma collected with an EDTA tube that was mixed with 300 µL TBARS acid reagent and was immediately followed by centrifuging at ≥12,000× *g* for 4 min to precipitate interfering proteins and other substances. Next, 150 µL of the supernatant was then added to a 96-well plate and mixed with 75 µL of TBA reagent. Optical density of each well was pre-read at 532 nm and re-read at the same wave length after the mixture was incubated for 3 h at 50 °C. Final absorbance for each well was measured by subtracting pre-reading from the final reading.

Total antioxidant capacity of plasma was measured using TAC assay kit (Abcam, Cambridge, CA, USA) according to the method described by Enseleit et al. in 2013 [23]. Briefly, Cu^2+^ is reduced to Cu^+^ by non-enzymatic antioxidants such as small molecules (uric acid, GSH, vitamins C and E, etc.) and proteins (albumin, transferrin, etc.). 100 µL diluted plasma sample collected with EDTA tubes were added into each well, which was followed by the addition of 100 µL Cu^2+^ working solution. The mixture was incubated for 1.5 h at room temperature and absorbance was read at 570 nm since the reduced Cu^+^ was chelated with a colorimetric probe reaching the peak absorbance of 570 nm. The unit of TAC concentration was expressed as Trolox Equivalent.

### 4.3. Analysis of Plasma PCB Levels (See Additional Details in Supplementary Section S2)

*Chemicals*: Internal standards and native standards for PCB congeners (purity = 99%) were purchased from Wellington Laboratories (Guelph, ON, Canada) and Ultra Scientific Analytical Solutions (North Kingstown, RI, USA). Stock solutions of each compound were prepared in nonane. Florisil was purchased from US Silica (Frederick, MD, USA). Other reagent chemicals were obtained from Fisher Scientific Canada (Ottawa, ON, Canada) and EMD Chemicals (Gibbstown, NJ, USA).

*Extraction of polychlorinated biphenyls*: The samples were extracted in accordance with AXYS method MLA-901 using an in house method based on EPA methods 8270D and 1668A and accredited by the Canadian Association for Laboratory Accreditation Inc., (CALA, Nepean, ON, Canada). Samples were received frozen and were stored at −20 °C prior to analysis. Each sample was allowed to thaw and then mixed well with a vortex mixer. For each sample, an accurately weighed subsample of approximately 3 mL was analyzed. An aliquot of labeled surrogate standard solutions (^13^C_12_ 2,2′,4,5,5′-PeCB (13C PCB-101), ^13^C_12_ 2,2′,3,4,4′,5,5′-HpCB (13C PCB-180), ^13^C_12_ 2,2′,3,3′4′,4′,5,5′-OcCB (13C PCB-194)) was added to each sample and allowed to equilibrate. Ethanol and saturated ammonium sulfate were added to the samples to precipitate the proteins followed by extraction of the analytes into hexane. Samples were cleaned up by adsorption column chromatography on florisil. An aliquot of internal standard (^13^C_12_ 2,2′,4,4′,5,5′-HxCB (13C PCB-153)) was added before instrumental analysis.

*Analysis of PCB by GC/MS*: Analysis of target analytes was performed on a low-resolution mass spectrometer (LRMS) equipped with a gas chromatograph (GC) detector operating in selective ion monitoring (SIM) mode acquiring two characteristic ions for each target and surrogate standard. Final concentrations were determined by isotope dilution/internal standard quantification procedure. For all target compounds, linear equations were determined from a 5 point calibration series. Reporting limits are the sample detection limit (SDL). SDLs are determined by converting the area equivalents to 3× the height of the chromatographic noise to a concentration. All PCBs were analyzed by AXYS Analytical Services Ltd. (Sidney, BC, Canada).

### 4.4. Statistical Analysis

Statistics was performed using the SPSS software (Ver. 21; IBM Corp., Armonk, NY, USA). Significance was set at *p* < 0.05. All values are reported as means ± standard deviation (SD) unless noted otherwise. Prior to the start of the study, subject number was determined from a power analysis based on our major outcome variables (body WL and serum PCBs). In particular, this analysis determined that *n* = 12 per diet group was required to detect a significant mean difference of WL (1.4 kg) between two different diet groups [3]. Sample size (*n* = 11) was required to detect the minimal difference (8 μg/kg) for PCB 180 [24]. Additionally, alpha was set to 0.05 in order to reach 90% power for one tailed analysis. Absolute changes in serum PCBs and anti-oxidant (%) change were calculated as the baseline values subtracted from the 12 and 64 weeks intervention values. All data was normally distributed with no violations of normality, thus parametric tests were performed. A 2 × 2 factor repeated measures ANOVA was performed for the WL (Phase 1; P-CR, weeks 0–12) (sex; M vs. F and time; CON vs. 12 weeks) and the WM (Phase 2; weeks 13–64) (group; mP-CR vs. HH and time; 13 weeks vs. 64 weeks) to determine main effects. Post hoc comparison (Bonferroni correction) was performed if there was an interaction. Additionally, Pearson’s correlation coefficient was used to assess for significant relationships between the percent changes in body composition (i.e., body weight, fat mass, body fat percentage, abdominal fat) and total PCBs and oxidative stress during WM Phases. Analyses were performed by assigning each intervention group a number code but were not performed blinded.

## 5. Results

### 5.1. Weight Loss Phase 1 (WL; P-CR; Weeks 0–12)

#### Subject Characteristics

Forty-three participants were recruited in this study. Three individuals did not adhere to the dietary protocol during the 12 weeks, and were dropped out from the study due to non-compliance (note: weight loss of dropped participants ranged from 2.0 to 5.0 kg and inclusion did not change statistical significance for any variable). Thus, 40 participants (21 men and 19 women) completed Phase 1. All participants met the inclusion criteria for overweight and obesity (BMI ≥ 27.5, % body fat >30%). Baseline characteristics of the participants are illustrated in Table 3.

### 5.2. Serum Toxins and Oxidative Stress Biomarkers during WL (Weeks 0–12)

Concentrations of serum PCBs and oxidative stress biomarkers following WL (Phase 1; P-CR) are shown in Table 4. Women and men responded similarly to the WL intervention and no differences existed for any of the variables (time *X* group, *p* > 0.05). A significant time effect was observed for individual PCBs 99, 118, 138, 153, 170, 180, 187, 194, and total PCBs (*p* = 0.01) as well as TBARS (*p* = 0.01) and TAC (*p* = 0.02). Plasma level of TBARS (lipid peroxidation) was significantly decreased (baseline: 0.24 ± 0.15 µM; 12 week: 0.18 ± 0.11 µM, *p* = 0.01) which coincided with an increase in TAC (baseline: 18.9 ± 2.6 nmol/mL; 12 week: 19.0 ± 2.3 nmol/mL, *p* = 0.02) (Table 4).

**Figure 4:**
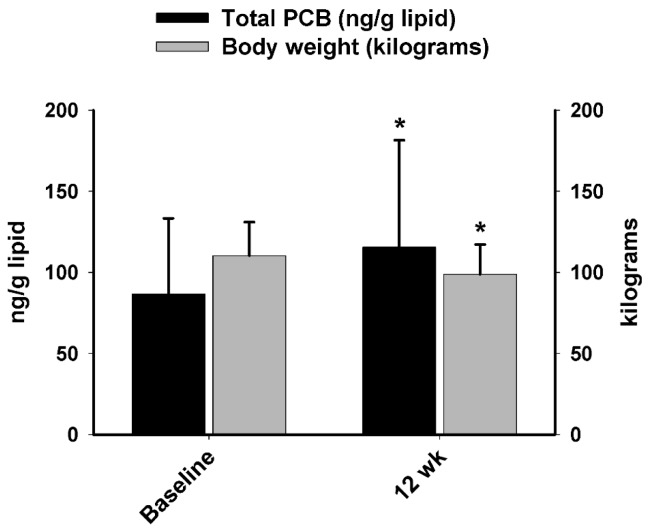
Total PCB concentration and body weight changes following Weight Loss Phase 1 (WL; P-CR, Weeks 0–12). * Significant difference compared to baseline (*p* < 0.01).

Additionally, PCBs were significantly increased after 12 weeks of WL (P-CR), which was accompanied with a significant body weight (11.5 kg) loss (*p* = 0.001) in the total group of women and men (Figure 4).

### 5.3. Weight Loss Maintenance Phase 2 (WM; mP-CR vs. HH; Weeks 13–64)

#### Subject Characteristics

Twenty-four participants completed the WM (Phase 2). Descriptive characteristics of participants at baseline (week-13, end of WL, Phase 1) are shown in Table 5. There were no significant differences between mP-CR and HH groups at the start of the WM (week 13).

### 5.4. Serum Toxins and Oxidative Stress Biomarkers during WM (Weeks 13–64)

**Figure 5:**
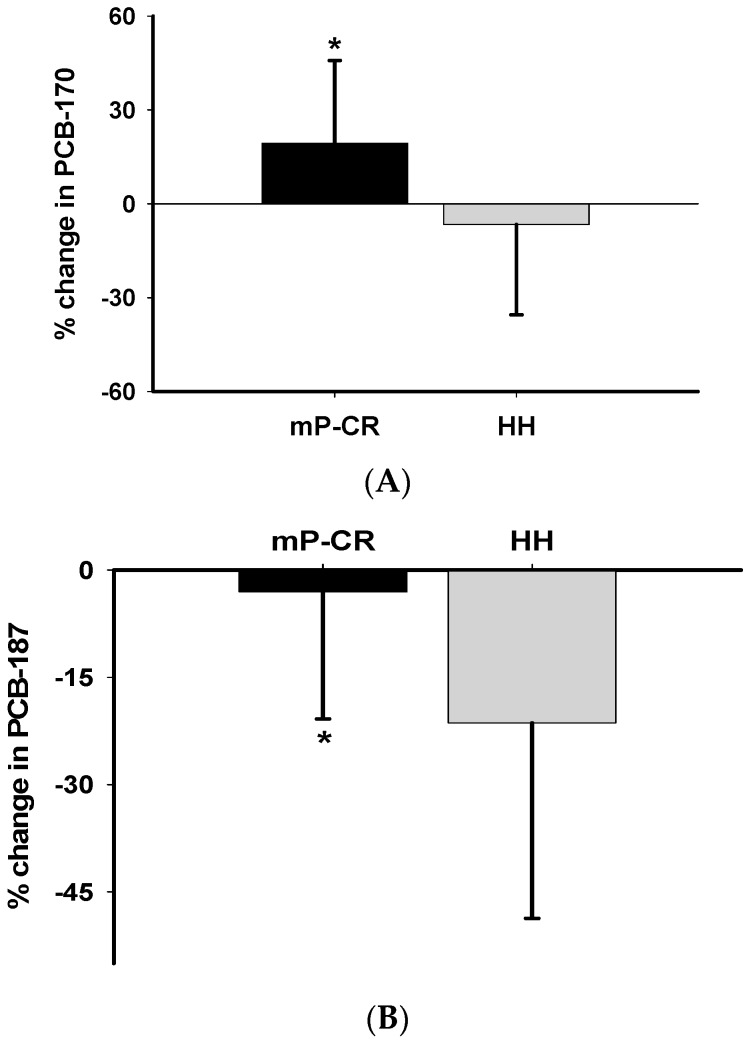
Percentage changes in PCB-170 (**A**) and PCB-187 (**B**) between mP-CR and HH groups following WM (Phase 2). * Significant group effect (mP-CR vs. HH; *p* < 0.05).

The percentage change of PCB 170 and PCB 187 was significantly higher in mP-CR compared to HH group (PCB 170: 19.31% ± 26.48% vs. −6.61% ± 28.88%, *p* = 0.02; PCB 187: −3.04% ± 17.78% vs. −21.4% ± 27.31%, *p* = 0.04) (Figure 5A,B).

Absolute changes in TBARS, TAC and PCBs between mP-CR and HH groups are shown in Table 6. There was a tendency (*p* > 0.05 to < 0.10) for PCBs 138, 153, 170, 180 and 187 to be significantly higher in mP-CR following WM compared to HH.

The current study found that changes in PCB 99, PCB 138, PCB 153, PCB 170, PCB 180, PCB 187, and PCB 194 were positively correlated with changes in TBARS (*r* = 0.57, *p* = 0.00, *r* = 0.45, *p* = 0.02, *r* = 0.47, *p* = 0.02; *r* = 0.44, *p* = 0.02; *r* = 0.42, *p* = 0.03; *r* = 0.50, *p* = 0.01; *r* = 0.40, *p* = 0.04, respectively). Interestingly, body composition parameters such as body weight, fat mass, body fat, and abdominal fat were negatively correlated with changes in TBARS (*r* = −0.46, *p* = 0.02; *r* = −0.51, *p* = 0.01; *r* = −0.58, *p* = 0.00; *r* = −0.53, *p* = 0.00, respectively) (Table 7).

Changes in circulating PCBs were inversely correlated with changes in body weight, fat mass, percentage of body fat, and abdominal fat, except individual PCBs such as PCB 74 and PCB 156 (Table 8).

## 6. Discussion

A major aim of the current study was to compare PCB and oxidative stress responses between obese men and women following a short-term (12 week) protein-pacing, caloric-restriction WL diet intervention. For WL Phase 1, we report most individual PCBs and total PCBs in serum increased similarly in men and women following the 12-week WL intervention, which was accompanied with significant weight and fat loss. This finding is consistent with previous studies [11,14,24,25,26,27,28,29,30] and extends them by directly comparing PCB changes in obese men and women following a P-CR diet. Another beneficial effect of P-CR is plasma oxidative stress measured by lipid peroxidation (TBARS) was decreased, whereas total antioxidant capacity (TAC) was boosted after WL (Phase 1). Obesity is commonly associated with increased chronic systemic oxidative stress which contributes to various metabolic diseases [31]. Thus, the decrease in oxidative stress biomarkers may be explained by decreased fat mass and increased antioxidant defense via the P-CR intervention. It is important to note, however, that our data are limited to TBARS and TAC. Future investigations should focus on evaluation of additional enzymatic antioxidant defense biomarkers.

For WM Phase 2 (weeks 13–64), we demonstrate that the: (1) absolute values and magnitude of decrease in many PCBs (PCBs 153, 180, 187, 194 and total PCBs) was significantly higher in HH than mP-CR and; (2) changes in PCBs are inversely related to changes in body weight/composition (i.e., body weight, fat mass, abdominal fat) and positively related to changes in biomarkers of oxidative stress (TBARS). Please note, the current study reports changes in PCBs and oxidative stress biomarkers during both weight loss (weeks 0–12) and weight maintenance (weeks 13–64) and not necessarily body composition changes as those have been published previously [6].

### 6.1. Weight Loss, Phase 1 (WL; P-CR, Weeks 0–12)

#### PCBs and Oxidative Stress Markers

PCBs were reported as organic pollutants which have toxic effects on the human body such as endocrine disruption and neurotoxicity [18]. As such, we performed an extensive blood panel (CBC, TSH, bilirubin, ALT, alkaline phosphatase) on all study participants and all values were within normal ranges. There was no difference at the baseline level (week-12) as well as the post intervention (week-64) for any of the hematology parameters between P-CR and HH participants (Table S1). Consistent with previous studies [17,24,25,30], we found levels of total PCBs in serum increased after 12 weeks of WL intervention in both sexes. Therefore, there are two aspects of WL in humans; the benefits include the decrease of adipose tissue mass whereas the possible harms are due to the increase of serum concentrations of lipophilic chemicals like PCBs.

However, there may be time differences for these two opposite aspects to reveal their biological effects in humans. In particular, the improved redox status of marked reduction of TBARS and an increase in TAC after Phase 1 WL period despite the increase of PCBs may not be surprising. For this perspective, possible benefits due to the decrease of adipose tissue mass may be more immediate, but possible harms due to increased lipophilic chemicals may need more time. WL via diet intervention is a novel approach to study the effect of PCBs on human health. Hitherto, few other studies investigated the relationship between oxidative stress and PCBs following WL in humans. In the present study, 12 weeks of a P-CR intervention suppressed lipid peroxidation (i.e., TBARS) and boosted total antioxidant capacity equally in obese men and women.

### 6.2. Weight Maintenance, Phase 2 (WM; mP-CR vs. HH, Weeks 13–64

#### PCBs and Oxidative Stress Markers

Longer-term (≥1 year) traditional follow-up diet interventions using ad libitum designs often show weight relapse and loss of beneficial effects resulting from initial rapid WL [1]. Another novel approach of our study was to investigate absolute changes in PCBs, and oxidative stress comparing mP-CR and traditional HH diets by 52 weeks (1 year) of follow up after an initial rapid 12 weeks WL (Phase 1). We separated this component as a different phase (Phase 2). Following 52 weeks of Phase 2 follow-up, the magnitude of decrease in PCB 153, PCB 180, PCB 187, PCB 194 and total TCBs was significantly higher in HH than mP-CR (Table 6). It is well known that elimination of accumulated organic pollutants in the human body is very challenging due to their persistent resistance to degradation [32]. Hence, the decrease in circulating PCBs is most likely due to reabsorption into adipose tissue. This is strongly supported by the inverse relationship between changes in body composition (i.e., fat mass, body weight, percentage of body fat, and abdominal fat) and circulating PCBs in the present study. In other words, body weight relapse is strongly correlated with decreased levels of PCBs in serum. However, the minimal decrease or even slight increase in PCBs in mP-CR after Phase 2 indicates mP-CR has an advantage in preventing the relapse of body weight compared to the traditional HH diet. To our best knowledge, we report for the first time a negative association between total PCBs and abdominal fat following 52-week diet (mP-CR, HH) interventions. This result indicates that increased PCBs in serum could be related to enhanced lipolysis from central localized fat following a diet intervention. As a result, further research should investigate this association more closely from a mechanistic perspective.

For Phase 2, our data revealed that mP-CR is more effective in preventing weight relapse than HH. However, it is unknown whether the relatively higher magnitude of increase in PCBs in mP-CR compared to HH may pose a health concern. It is well established in animal studies that PCBs exhibit adverse effects on endocrine disruption, cancer and reproduction [17,18,33,34]. The precise mechanism for the PCB-induced detrimental effect are continuing to be fully elucidated [35,36,37] and oxidative stress induced by PCBs is associated with this toxic manifestation in numerous animal studies [38,39,40,41,42,43,44]. Additionally, to substantiate the previous finding, our results revealed a positive association between changes in circulating oxidative stress (i.e., TBARS) and PCBs following Phase 2. Interestingly, no significant difference was found in terms of oxidative stress between mP-CR and HH group. This suggests that mP-CR had an advantage in preventing the reverse of improvement gained after initial rapid WL in the absence of elevated oxidative stress associated with the increase in circulating PCBs. It is important to highlight the P-CR (and mP-CR) diet was abundant in antioxidant-rich nutrients which may have potentially counterbalanced the increase in oxidative stress induced by the release of circulating PCBs and may have contributed to the enhanced antioxidant capacity in these participants. An alternative explanation may be that the increase in serum PCB concentrations was too low to induce any oxidative stress.

Additionally, the greater WM and higher serum PCBs in mP-CR compared to HH without further worsening oxidative stress may be due to the higher antioxidant component in the diet. Nevertheless, the harmful effects induced by the increased lipophilic chemicals may need more time. Therefore, this might explain why we observed no statistical significance in TBARS and TAC between mP-CR and HH groups despite the relatively high magnitude of increase in PCBs in mP-CR compared to HH.

### 6.3. Strengths and Limitations

Several noteworthy novelties and strengths of the current study are: (a) direct comparison between obese men and women for PCB and oxidative stress response to a commonly prescribed weight loss diet (P-CR); (b) exploring the relationship of PCBs and oxidative stress in the context of WL; (c) inclusion of different phases (WL; WM) that include distinct baselines for direct comparison within each intervention; (d) Exploring the interrelated role of WL, plasma toxins, and oxidative stress by comparing a mP-CR diet with traditional HH; (e) close supervision of the nutritional and physical activity level of all participants during the WL and WM phases; and (f) familiarization and normalization of each measurement and laboratory procedures.

It is equally important to address several limitations within the current study, including: (a) obesity-related abnormalities in metabolic status may have impacted the response to the WL interventions and a more thorough assessment of metabolic status (e.g., triglyceride level, blood pressure, blood glucose levels, cholesterol levels, resting metabolic rate, etc.) could have been obtained; (b) apparent differences in completion rates existed between the groups (9 of 19 mP-CR vs. 4 of 18 HH participants were excluded from analysis) during the maintenance phase due to drop-out, scheduling conflicts, and non-compliance (see Table S3) of participants. Importantly, inclusion of non-compliant participants resulted in only a trend for mP-CR to confer additional benefit over HH following weight maintenance (mP-CR, 98.9 ± 16.5 vs. 101.8 ± 20.6; HH, 94.5 ± 13.7 vs. 100.6 ± 14.2 kg, *p* = 0.09) and warrants further investigation; (c) it is well known that long-term weight loss studies are associated with higher drop-out rates compared to short-term interventions and thus, participants completing Phase 2 were likely highly motivated and committed to the study, whereas those less motivated dropped out, thus reducing the possibility of false positive findings. This unlikely affected the outcomes of our findings because baseline measurements at the start of Phase 2 (WM) were similar between groups; and (d) lastly, participants in this study received weekly subject-investigator contact to facilitate compliance which may have increased the risk of investigator bias. Individuals choosing to adopt this specific nutritional regimen on their own may not experience the same benefits as those achieved in the current study.

## 7. Conclusions

In summary, a 12-week P-CR diet effectively induced WL, favorably altered redox status, and increased circulating levels of serum PCBs to a similar extent in both obese women and men. Moreover, we provide novel findings showing that a 52-week mP-CR intervention prevents weight relapse in the absence of adverse effects (i.e., increased oxidative stress, abnormal blood panel) induced by elevated PCBs compared to a traditional HH diet. The current study demonstrates that a P-CR nutritional intervention, when coupled with close observation of compliance and dietary counseling, should be regarded as an effective dietary plan to mobilize stored PCBs and improve redox status. Future studies should investigate the mechanistic pathway (e.g., oxidative stress mediated pathway) of WL-induced PCB elevations and subsequent elimination from the body. Effective strategies such as the combination of proper nutrition and exercise should be designed in order to maintain the improved body composition and redox status along with the mobilization of stored PCBs during WL.

## Figures and Tables

**Table 1:** Sample menus and meal timing for women and men during WL phase (Protein-pacing/caloric-restriction, P-CR; Weeks 0–12). Menus were isocaloric for all women and men, respectively.

Variable	Women (1200 kcals/Day)	Men (1500 kcals/Day)
Breakfast (06:00–08:00)	Liquid protein meal IsaLean^®^; 240 kcals, 24 g protein, 24 g carbohydrate, 5 g fat; Caffeine beverage e+^®^; Multi-Vitamin/Mineral Ageless Essentials^®^; Anti-Oxidants Ionix^®^; 20 kcals	Liquid protein meal IsaLean^®^; 240 kcals, 24 g protein, 24 g carbohydrate, 5 g fat; Caffeine beverage e+^®^; Multi-Vitamin/Mineral Ageless Essentials^®^; Anti-Oxidants Ionix^®^; 20 kcals
Lunch (11:00–13:00)	Liquid protein meal IsaLean^®^; 240 kcals, 24 g protein, 24 g carbohydrate, 5 g fat	Liquid protein meal IsaLean^®^; 240 kcals, 24 g protein, 24 g carbohydrate, 5 g fat
Mid-Afternoon snack (14:00–16:00)		Greek yogurt, fruit; 150 kcals, 20 g protein; 12 g carbohydrate; 4 g fat
Dinner (17:00–19:00)	Fish/Poultry/Beef, fresh vegetables, chopped nuts, dried fruit, olive oil, milk; 450 kcals, 25 g protein; 50 g carbohydrate; 17 g fat	Fish/Poultry/Beef, fresh vegetables, chopped nuts, dried fruit, olive oil, milk; 600 kcals, 25 g protein; 69 g carbohydrate; 25 g fat
Evening snack (21:00–22:00)	Protein bar IsaLean^®^; 250 kcals, 18 g protein; 27 g carbohydrate; 9 g fat	Protein bar IsaLean^®^; 250 kcals, 18 g protein; 27 g carbohydrate; 9 g fat

**Table 2:** Composition of intermittent-fasting day diet during WL Phase 1 ^a^.

Food/Supplement	Frequency	Quantity
Whole-food high-protein snack	1/day	100 or 200 kcal for females and males, respectively
Anti-oxidant plant-based powder ^b^	6/day	120 kcal total
Low-glycemic protein wafers ^c^	3/day	90 kcal total
Micronutrient supplement ^d^	2/day	Contains vitamins, minerals, phytonutrients, antioxidants, and essential fatty acids
Plant-based herbal adaptogen powder ^e^	1/day	20 kcal total

^a^ During WL Phase 1, participants performed one day of IF per week; total energy intake of 330 kcal/day for women and 430 kcal/day for men. All supplements were provided by Isagenix LLC, Chandler, AZ, USA; ^b^ Cleanse for Life^®^; ^c^ Snacks™; ^d^ Ageless Essentials with Product B, AM & PM^®^, consumed on IF and non-IF days; ^e^ Ionix Supreme^®^, consumed on IF and non-IF days.

**Table 3:** Baseline (Week 0) characteristics of study participants for WL (Phase 1).

Variable	Men (*n* = 21)	Women (*n* = 19)	Total (*n* = 40)
Age (years)	46.1 ± 6.7	49.4 ± 10.9	47.6 ± 9.5
Height (cm)	178.9 ± 7.9	163.0 ± 4.5	171.1 ± 10.4
Weight (kg)	120.1 ± 22.0	99.5 ± 12.3	110.3 ± 20.6
Body fat (%)	40.3 ± 6.1	51.0 ± 3.9	45.4 ± 7.4
Body mass index (kg/m^2^)	37.5 ± 6.9	37.4 ± 4.8	37.4 ± 5.9
Systolic blood pressure (mmHg)	128.7 ± 11.7	125.0 ± 13.5	127.0 ± 12.5
Diastolic blood pressure (mmHg)	81.6 ± 11.0	76.9 ± 11.0	79.4 ± 11.1
Resting heart rate (bpm)	65.0 ± 8.7	64.7 ± 11.7	64.8 ± 10.1

All values are expressed as mean ± SD.

**Table 4:** Serum PCB congeners and oxidative stress biomarkers following WL (P-CR) intervention (Phase 1).

Variable	Men (*n* = 21)	Women (*n* = 19)	Total (*n* = 40)
*PCB congeners (ng/g lipid)*			
PCB 74			
Baseline	6.1 ± 2.5 (5.5)	8.2 ± 5.3 (6.6)	7.1 ± 4.2 (6.1)
Week 12	6.3 ± 3.5 (5.6)	8.9 ± 6.7 (5.7)	7.5 ± 5.4 (5.6)
Time (*p*-value)	0.38	0.35	0.32
PCB 99			
Baseline	3.6 ± 1.2 (3.3)	4.3 ± 2.2 (4.6)	3.9 ± 2.0 (3.7)
Week 12	4.2 ± 2.5 (4.4)	5.8 ± 2.3 (5.8)	4.9 ± 2.5 (4.6)
Time (*p*-value)	0.09	0.01 *	0.01 *
PCB 118			
Baseline	5.5 ± 4.0 (4.4)	6.6 ± 4.6 (4.6)	6.0 ± 4.3 (4.5)
Week 12	9.9 ± 7.9 (6.7)	10.3 ± 6.8 (9.4)	10.1 ± 7.3 (8.6)
Time (*p*-value)	0.01 *	0.01 *	0.01 *
PCB 138			
Baseline	15.7 ± 9.0 (12.7)	17.1 ± 12.4 (11.2)	16.3 ± 10.6 (12.5)
Week 12	20.9 ± 12.4 (20.0)	23.9 ± 16.4 (15.2)	22.3 ± 14.4 (18.0)
Time (*p*-value)	0.01 *	0.02 *	0.01 *
PCB 146			
Baseline	3.1 ± 3.1 (2.3)	2.2 ± 1.7 (1.9)	2.7 ± 2.6 (2.0)
Week 12	3.2 ± 2.1 (2.2)	3.0 ± 2.1 (2.4)	3.1 ± 2.1 (2.3)
Time (*p*-value)	0.42	0.01 *	0.13
PCB 153			
Baseline	19.0 ± 10.5 (15.4)	19.1 ± 14.1 (14.1)	19.1 ± 12.1 (15.0)
Week 12	26.8 ± 15.9 (24.5)	27.6 ± 18.9 (18.9)	27.1 ± 17.2 (22.2)
Time (*p*-value)	0.01 *	0.01 *	0.01 *
PCB 156			
Baseline	5.1 ± 2.7 (4.7)	3.6 ± 3.3 (2.4)	4.4 ± 3.0 (3.6)
Week 12	3.2 ± 1.7 (2.8)	4.5 ± 3.7 (3.3)	3.9 ± 2.9 (3.0)
Time (*p*-value)	0.01 *	0.04 *	0.14
PCB 170			
Baseline	5.7 ± 3.3 (5.0)	5.1 ± 3.4 (4.3)	5.4 ± 3.3 (4.5)
Week 12	6.6 ± 3.7 (6.2)	6.4 ± 4.3 (4.9)	6.5 ± 3.9 (5.8)
Time (*p*-value)	0.02 *	0.02 *	0.01 *
PCB 180			
Baseline	15.4 ± 8.5 (15.4)	13.1 ± 9.2 (11.0)	14.3 ± 8.8 (13.7)
Week 12	21.1 ± 12.8 (21.9)	19.9 ± 13.2 (17.4)	20.6 ± 12.8 (19.1)
Time (*p*-value)	0.01 *	0.01 *	0.01 *
PCB 187			
Baseline	4.3 ± 2.6 (3.2)	3.7 ± 2.1 (3.1)	4.0 ± 2.3 (3.1)
Week 12	5.6 ± 3.7 (4.5)	4.6 ± 3.1 (3.2)	5.1 ± 3.4 (4.6)
Time (*p*-value)	0.01 *	0.04 *	0.01 *
PCB 194			
Baseline	3.7 ± 2.4 (3.4)	2.9 ± 1.5 (2.8)	3.3 ± 2.0 (3.0)
Week 12	4.5 ± 3.4 (4.1)	3.9 ± 2.2 (4.1)	4.3 ± 2.9 (4.1)
Time (*p*-value)	0.02 *	0.02 *	0.01 *
Total PCB			
Baseline	87.3 ± 37.9 (82.2)	86.0 ± 53.9 (60.3)	86.7 ± 45.6 (78.1)
Week 12	112.5 ± 59.8 (98.5)	119.1 ± 76.7 (87.6)	115.6 ± 65.9 (97.1)
Time (*p*-value)	0.01 *	0.01 *	0.01 *
*Markers of oxidative stress*			
TBARS (µM)			
Baseline	0.27 ± 0.17	0.20 ± 0.13	0.24 ± 0.15
Week 12	0.19 ± 0.10	0.15 ± 0.11	0.18 ± 0.11
Time (*p*-value)	0.03 *	0.03 *	0.01 *
TAC (nmol/mL)			
Baseline	19.2 ± 2.8	18.6 ± 2.4	18.9 ± 2.6
Week 12	20.7 ± 2.6	19.0 ± 2.3	19.9 ± 2.3
Time (*p*-value)	0.03 *	0.24	0.02 *

All values are expressed as mean ± SD and median ( ). * Significantly different from baseline (*p* < 0.05).

**Table 5:** Baseline (Week 13) characteristics of study participants for WM (Phase 2).

Variable	mP-CR (*n* = 10)	HH (*n* = 14)	Total (*n* = 24)
Age (years)	50.9 ± 9.7	50.0 ± 6.8	50.4 ± 7.9
Height (cm)	169.6 ± 9.8	170.2 ± 12.4	169.9 ± 11.2
Weight (kg)	90.4 ± 7.5	95.1 ± 14.0	93.1 ± 11.8
Body fat (%)	40.2 ± 8.3	39.2 ± 8.7	39.6 ± 8.4
Body mass index (kg/m^2^)	31.6 ± 2.6	33.1 ± 4.7	32.5 ± 4.0
Systolic blood pressure (mmHg)	114.0 ± 10.3	110.9 ± 9.2	112.2 ± 9.6
Diastolic blood pressure (mmHg)	68.9 ± 8.2	69.3 ± 7.4	69.1 ± 7.5
Resting heart rate (bpm)	57.4 ± 8.8	58.7 ± 10.1	58.2 ± 9.4

All values are expressed as mean ± SD.

**Table 6:** Oxidative stress markers, body composition, and PCBs following WM (Phase 2).

Variable	mP-CR (*n* = 10)	HH (*n* = 14)	Group × Time (*p*-Value)
*Markers of oxidative stress*			
TBARS (μM)			
Baseline (week-13)	0.14 ± 0.1	0.18 ± 0.1	
Week-64	0.39 ± 0.2	0.35 ± 0.1	0.33
Time (*p*-value)	0.09	0.02 *	
TAC (nmol/mL)			
Baseline (week-13)	19.1 ± 0.8	20.6 ± 0.6	
Week-64	15.2 ± 1.2	17.3 ± 1.7	0.41
Time (*p*-value)	0.04 *	0.07	
*Body weight*			
Body Weight (kg)			
Baseline (week-13)	90.4 ± 2.4	95.1 ± 3.7	
Week-64	91.1 ± 3.5	100.6 ± 3.9	0.04 **
Time (*p-*value)	0.33	0.01 *	
*PCB congeners (ng/g lipid)*			
PCB 74			
Baseline (week-13)	8.0 ± 1.3 (6.7)	9.1 ± 2.0 (5.7)	
Week-64	6.9 ± 1.0 (7.3)	6.4 ± 1.1 (5.4)	0.28
Time (*p*-value)	0.13	0.10	
PCB 99			
Baseline (week-13)	6.4 ± 0.9 (4.4)	5.8 ± 0.6 (5.9)	
Week-64	5.0 ± 0.9 (3.4)	4.1 ± 0.5 (4.0)	0.37
Time (*p*-value)	0.04 *	0.01 *	
PCB 118			
Baseline (week-13)	11.3 ± 2.0 (9.4)	10.8 ± 1.6 (10.2)	
Week-64	8.4 ± 1.7 (7.1)	6.5 ± 0.9 (6.6)	0.27
Time (*p*-value)	0.01 *	0.01 *	
PCB 138			
Baseline (week-13)	26.6 ± 4.9 (15.9)	28.4 ± 3.1 (26.7)	
Week-64	26.9 ± 6.4 (17.5)	21.4 ± 2.6 (20.4)	0.10
Time (*p*-value)	0.46	0.06	
PCB 146			
Baseline (week-13)	3.6 ± 0.7 (2.2)	4.0 ± 0.4 (3.9)	
Week-64	3.9 ± 0.7 (3.0)	3.8 ± 0.4 (3.6)	0.24
Time (*p*-value)	0.21	0.32	
PCB 153			
Baseline (week-13)	33.0 ± 6.4 (19.9)	34.7 ± 3.3 (35.4)	
Week-64	32.3 ± 8.4 (20.8)	25.6 ± 3.1 (24.5)	0.08
Time (*p*-value)	0.41	0.03 *	
PCB 156			
Baseline (week-13)	4.2 ± 0.9 (2.8)	4.7 ± 0.5 (4.5)	
Week-64	4.4 ± 1.0 (3.8)	4.5 ± 0.5 (4.1)	0.30
Time (*p*-value)	0.32	0.36	
PCB 170			
Baseline (week-13)	6.6 ± 1.2 (4.9)	8.4 ± 0.8 (8.4)	
Week-64	8.1 ± 1.9 (6.3)	7.7 ± 1.0 (6.7)	0.06
Time (*p*-value)	0.06	0.23	
PCB 180			
Baseline (week-13)	22.6 ± 4.0 (20.6)	27.2 ± 3.0 (26.3)	
Week-64	21.0 ± 5.2 (16.7)	19.7 ± 2.7 (18.2)	0.07
Time (*p*-value)	0.16	0.01 *	
PCB 187			
Baseline (week-13)	6.1 ± 1.2 (5.6)	6.8 ± 0.8 (6.8)	
Week-64	5.8 ± 1.3 (5.1)	5.1 ± 0.8 (4.4)	0.06
Time (*p*-value)	0.31	0.01 *	
PCB 194			
Baseline (week-13)	4.6 ± 0.7 (4.3)	5.8 ± 0.9 (4.6)	
Week-64	4.3 ± 0.9 (3.8)	4.6 ± 0.7 (4.0)	0.13
Time (*p*-value)	0.19	0.02 *	
Total PCB			
Baseline (week-13)	125.7 ± 21.7 (92.3)	145.5 ± 14.1 (142.8)	
Week-64	119.9 ± 27.3 (92.4)	109.3 ± 11.6 (102.7)	0.11
Time (*p*-value)	0.29	0.04 *	

All values are expressed as mean ± SE and median ( ). * significantly different from baseline (week-13). ** Significant difference between mP-CR and HH groups.

**Table 7:** Pearson correlation coefficients for the percent change in oxidative stress, PCBs, and body composition following the WM (Phase 2).

Variable	TBARs		TAC	
*PCBs*	*r* Value	*p* Value	*r* Value	*p* Value
PCB 74	0.03	0.46	0.19	0.20
PCB 99	0.57	*p* < 0.01 **	−0.12	0.30
PCB 118	0.21	0.18	0.12	0.30
PCB 138	0.45	0.02 *	0.20	0.18
PCB 146	0.01	0.48	0.35	0.06
PCB 153	0.47	0.02 *	0.18	0.22
PCB 156	−0.02	0.47	0.21	0.18
PCB 170	0.44	0.02 *	−0.21	0.18
PCB 180	0.42	0.03 *	0.11	0.32
PCB 187	0.50	0.01 *	0.01	0.48
PCB 194	0.40	0.04 *	0.05	0.42
Total PCB	0.35	0.06	0.21	0.17
***Body composition***				
Body weight	−0.46	0.02 *	−0.10	0.33
Fat mass	−0.51	0.01 *	−0.16	0.24
Body fat	−0.58	*p* < 0.01 **	−0.17	0.23
Abdominal fat	−0.53	*p* < 0.01 **	−0.06	0.39

* Significantly correlated (*p* < 0.05). ** Significantly correlated (*p* < 0.01).

**Table 8:** Pearson correlation coefficients for the percent change in body composition and PCBs following the WM Phase 2 study.

Variable	Body Weight		Fat Mass		Body Fat		Abdominal Fat	
*PCBs*	*r* Value	*p* Value	*r* Value	*p* Value	*r* Value	*p* Value	*r* Value	*p* Value
PCB 74	−0.34	0.06	−0.38	0.04 *	−0.34	0.07	−0.29	0.09
PCB 99	−0.43	0.02 *	−0.41	0.03 *	−0.39	0.04 *	−0.30	0.08
PCB 118	−0.56	*p* < 0.01 **	−0.54	*p* < 0.01 **	−0.45	0.02 *	−0.45	0.02 *
PCB 138	−0.67	*p* < 0.01 **	−0.65	*p* < 0.01 **	−0.56	*p* < 0.01 **	−0.53	*p* < 0.01 **
PCB 146	−0.37	0.04 *	−0.45	0.02 *	−0.45	0.02 *	−0.41	0.03 *
PCB 153	−0.68	*p* < 0.01 **	−0.67	*p* < 0.01 **	−0.59	*p* < 0.01 **	−0.57	*p* < 0.01 **
PCB 156	−0.06	0.39	−0.19	0.20	−0.30	0.10	−0.15	0.25
PCB 170	−0.59	*p* < 0.01 **	−0.55	*p* < 0.01 **	−0.46	0.02 *	−0.49	*p* < 0.01 **
PCB 180	−0.73	*p* < 0.01 **	−0.71	*p* < 0.01 **	−0.62	*p* < 0.01 **	−0.61	*p* < 0.01 **
PCB 187	−0.71	*p* < 0.01 **	−0.67	*p* < 0.01 **	−0.56	*p* < 0.01 **	−0.50	*p* < 0.01 **
PCB 194	−0.51	*p* < 0.01 **	−0.47	0.01 *	−0.38	0.05 *	−0.34	0.05
Total PCB	−0.62	*p* < 0.01 **	−0.64	*p* < 0.01 **	−0.59	*p* < 0.01 **	−0.54	*p* < 0.01 **

* Significant correlated (*p* < 0.05). ** Significantly correlated (*p* < 0.01).

## Supplementary Materials

The following are available online at www.mdpi.com/1660-4601/14/1/59/s1, S1: Hematology parameters at baseline and post-intervention for Weight Maintenance (Phase 2); S2: Supplemental PCB Analytical Procedures; S3: Weight Loss and PCB Data from Non-Compliant Participants during WM (Phase 2).

## Abbreviations

The following abbreviations are used in this manuscript:

IF	intermittent fasting
P-CR	protein-pacing, caloric restriction
mP-CR	modified protein-pacing, caloric restriction
HH	heart healthy
WL	weight loss
WM	weight maintenance
PCBs	polychlorinated biphenyls
TBARS	thiobarbituric acid reactive substances
TAC	total antioxidant capacity
