# Supplementary material for: Serum Polychlorinated Biphenyls Increase and Oxidative Stress Decreases with a Protein-Pacing Caloric Restriction Diet in Obese Men and Women
Source: Int J Environ Res Public Health. 2017 Jan 10;14(1):59. doi: 10.3390/ijerph14010059 (PMC5295310; doi:10.3390/ijerph14010059)
Supplement: Supplementary file 1 [file ijerph-14-00059-s001.pdf]

Supplementary Materials: Serum Polychlorinated Biphenyls Increase and Oxidative Stress Decreases with a Protein-Pacing Caloric Restriction Diet in Obese Men and Women

Feng He, Li Zuo, Emery Ward and Paul J. Arciero

S1. Hematology parameters at baseline and post-intervention for Weight Maintenance (Phase 2) (Table S1).

Table S1. Hematology parameters at baseline and post-intervention for Weight Maintenance (Phase 2).

	Timeline	Group	WBC	RBC	Hematocrit	TSH	Bilirubin	ALT	Alkaline Phosphatase
a	***Baseline***	HH (n = 13)	5.50 ± 1.09	4.87 ± 0.32	42.69 ± 1.73	1.74 ± 0.85	0.54 ± 1.14	35.00 ± 9.99	90.15 ± 24.45
	(week-12)	mP-CR (n = 16)	6.43 ± 1.49	4.73 ± 0.49	41.48 ± 3.45	2.65 ± 1.82	0.73 ± 0.64	44.56 ± 26.25	95.63 ± 21.65
		p-value	0.07	0.39	0.26	0.11	0.31	0.23	0.53
	Post-intervention	HH(n = 7)	5.51 ± 1.20	14.42 ± 0.40	42.14 ± 1.49	1.70 ± 0.71	0.53 ± 0.09	36.43 ± 14.63	86.29 ± 16.10
	(week-64)	mP-CR (n = 9)	6.24 ± 1.15	14.04 ± 1.32	41.12 ± 3.59	2.92 ± 2.24	0.57 ± 0.25	41.11 ± 20.25	92.89 ± 18.90
		p-value	0.24	0.54	0.49	0.19	0.71	0.62	0.47

(a) All values are expressed as mean ± SD. No significant difference was found between mP-CR and HH groups. p was set at 0.05.

S2. Supplemental PCB Analytical Procedures.

Serum: Allow serum samples to thaw at room temperature prior to subsampling for analysis. Homogenize by vortex mixing. Samples must be extracted within 24 h of thawing.

Preparation of Standard Solutions: The analysis of PCBs by low resolution GC/MS requires the use of the surrogate, recovery, and calibration standards described below. Details of the preparation, composition and validation of concentrations of these standards are described below.

Surrogate Standard: Samples are spiked with a suite of isotopically labelled surrogate standards prior to extraction procedures. The surrogate standard solution is prepared to have the nominal concentrations of surrogates presented in Supplemental Table S2. An aliquot of surrogate standard solution is added to each sample prior to extraction. Typically, 20 μL, equivalent to approximately 1 ng of each labelled congener, is added to the sample. The amount of surrogate added is dependent upon sample size, final extract volume, and concentration of analytes in the sample and may be adjusted accordingly.

Recovery Standard: Samples extracts are spiked with an isotopically labelled recovery standard prior to instrumental analysis. The recovery standard solution is prepared to have the nominal concentrations of recovery standard presented in Table S2. An aliquot of recovery standard is added to the final extract just prior to instrumental analysis. The amount of recovery standard added is designed to match the amount of surrogate in the final extract.

Calibration Solutions: A series of five calibration solutions containing native analytes, labelled surrogates standards, and labelled recovery standards are used to demonstrate linearity of the analytical instrument. The calibration solutions are prepared to have the nominal concentrations presented in Table S3. The concentration of the native analytes in the solutions varies to encompass the working range of the instrument, while the concentrations of the surrogates and recovery standards remain constant. 'Bracketing' calibration is performed by analyzing a mid-level solution at the beginning and end of analytical runs, with a minimum frequency of every 13 h.

Authentic Spiking Standard: The authentic spiking solution is prepared to have the nominal concentrations presented in Table S2. An aliquot of authentic spiking solution is added to an unspiked reference material (USM) to prepare a spiked reference sample as a QC sample. Typically, a 20-μL aliquot, equivalent to 1 ng of each target analyte is added to the matrix.

Solvent Blank: During the analysis of a batch of samples, a solvent blank is analyzed after the calibration solution to monitor carryover from the previous injection. The solvent blank is prepared in nonane, and contains PCB 157 as a sensitivity standard to monitor the instrument detection limits.

Table S2. Analysis of PCB congeners by GC/LRMS. Nominal concentrations of authentic, surrogate and recovery standard solutions (ng/mL).

Compound Name	Concentration in Authentic Standard Solution (ng/mL)
PCB 74	50
PCB 99	50
PCB 118	50
PCB 138	50
PCB 146	50
PCB 153	50
PCB 156	50
PCB 170	50
PCB 180	50
PCB 187	50
PCB 194	50
HCB	80

Labelled Surrogates	Concentration in Surrogate Standard Solution (ng/mL)
$^{13}C_{12}$-PCB 101	50
$^{13}C_{12}$-PCB 180	50
$^{13}C_{12}$-PCB 194	50
$^{13}C_{6}$-HCB	160
**Recovery Standard**	**Concentration in Recovery Standard Solution (ng/mL)**
$^{13}C_{12}$-PCB 153	100

Table S3. Analysis of PCB congeners by GC/LRMS nominal concentrations of calibration solutions (ng/mL).

Compound Name	Calibration Standards				
	Level A	Level B	Level C (Mid-Level)	Level D	Level E
PCB 74	2	10	25	100	500
PCB 99	2	10	25	100	500
PCB 118	2	10	25	100	500
PCB 138	2	10	25	100	500
PCB 146	2	10	25	100	500
PCB 153	2	10	25	100	500
PCB 156	2	10	25	100	500
PCB 170	2	10	25	100	500
PCB 180	2	10	25	100	500
PCB 187	2	10	25	100	500
PCB 194	2	10	25	100	500
**Surrogate Standard**					
$^{13}C_{12}$-PCB 101	25	25	25	25	25
$^{13}C_{12}$-PCB 180	25	25	25	25	25
$^{13}C_{12}$-PCB 194	25	25	25	25	25
$^{13}C_{6}$-HCB	80	80	80	80	80
**Recovery Standard**					
$^{13}C_{12}$-PCB 153	25	25	25	25	25

Quality Assurance/Quality Control: All samples are analyzed in batches. The composition of a batch is detailed on a batch sheet. Each batch has the following composition:

- Batch Size—Each batch consists of up to twenty test samples and additional QC samples.
- Blanks—One procedural blank is analyzed for each batch. A bovine calf serum previously shown to have no detectable PCBs (<0.03 ng/g per congener) is used for the blank matrix.
- Duplicates—Duplicate samples are analyzed as required by contract. In the event that there is insufficient serum available for a duplicate analysis a second reference sample (see next bullet) will be added to the batch.
- Reference Samples—Spiked samples are relied on to demonstrate the accuracy of the data. Serum is spiked with authentic, surrogate and recovery standards and analyzed alongside client samples. At least one spike is run in every batch of samples. A bovine calf serum previously shown to have no detectable PCBs (<0.03 ng/g per congener) is used for the matrix.
- Surrogate/Authentic/Recovery (SAR) solution is an optional internal diagnostic test that may be prepared and analyzed with a batch.

The quality control limits for duplicate samples, procedural blanks, reference samples, surrogate recoveries, and detection limits are specified in Tables S4–S6 in the following pages of this document.

Table S4. Analysis of PCB congeners by GC/LRMS method: QA/QC specifications.

MATRIX	Serum, RL	Procedural Blank Level	Acceptable Matrix Spike
**Analyte**	**ng/g**	**ng**	**% Recovery**
PCB 74	0.03	ND	75–125
PCB 99	0.03	ND	75–125
PCB 118	0.03	ND	75–125
PCB 138	0.03	ND	75–125
PCB 146	0.03	ND	75–125
PCB 153	0.03	ND	75–125
PCB 156	0.03	ND	75–125
PCB 170	0.03	ND	75–125
PCB 180	0.03	ND	75–125
PCB 187	0.03	ND	75–125
PCB 194	0.03	ND	75–125
Typical sample size:	3 g		
Typical final extract volume, μL	40		
Authentic Spike (ng)	1		

ND ≤ 0.03 ng/g.

Table S5. Surrogate standard recovery ranges.

Surrogate Standard	% Recovery Ranges
RECOVERIES:	
$^{13}C_{12}$-PCB 101	50–120
$^{13}C_{12}$-PCB 180	50–120
$^{13}C_{12}$-PCB 194	50–120

Table S6. Quality control specifications.

QC Parameter	Specification
Analysis Duplicate	40% RPD for analytes above 10× detection limit.
Procedural Blank	See above; concentrations in the laboratory blanks must be non-detect with a detection limit of ≤0.03 ng/g.
Matrix Spike Recovery	Concentrations of spiked congeners must fall within ±25 of expected values.
Surrogate Std. Recovery	See above.
Instrument Sensitivity	S:N ratio must be at least 3:1 for all congeners in A cal.
Instrument Linearity	Linearity is determined by at least a 5-point calibration with a relative standard deviation of the RRF's ≤20%.
Bracketing Calibration	RRFs for the opening and closing calibration standards over a 13 h period must agree to within ±15% of the mean, i.e., ≤30 RPD. Note that 30 RPD is equivalent to 21.2% RSD.
Chromatogram Quality	Target peaks must fall within ±3 s of the predicted retention times.
Analyte/Surrogate Ratios	Target response must be within the calibrated range of the instrument. Coders may use data from more than one chromatogram to get the responses in the calibrated range.
Ion Ratios	Ion ratios must fall within ±20% of the theoretical values for positive identification of all targets in the calibration standards and samples.

Serum Extraction Procedure: This extraction procedure is applicable to the matrices and sample size listed below (Table S7).

Table S7. Applicable extraction matrix sample size.

Matrix	Sample Size
Blood Serum	3–5 g

Accurately weigh a sample of serum to at least three significant figures into a 250 mL round bottom flask. Add an aliquot of surrogate standards and allow to equilibrate for 15 min.

1. For serum samples up to 10 g, add 10 mL ethanol, 50 mL hexane and 10 mL saturated ammonium sulphate to the sample and extract by shaking on the shaker table for 30 min.
2. Decant the hexane layer into a 250 mL separatory funnel.
3. Add 50 mL hexane to the aqueous layer in the round bottom flask and shake the mixture for 30 min on a shaker table. Decant the hexane into the separatory funnel containing the first hexane extraction. Discard the bottom aqueous layer.
4. Backwash the hexane extract by shaking with ultra-pure water (2 × 50 mL) to remove any residual ethanol.
5. Drain the hexane extract into a 250 mL Erlenmeyer flask and dry with anhydrous sodium sulphate. Quantitatively transfer the extract with hexane rinses into a clean round-bottom flask or a "Syncore Analyst" evaporator tube.

Concentrate the extract to 1 mL by rotary evaporation or using a "Syncore Analyst" evaporator. The extract is ready for the column cleanup procedure.

Cleanup Procedures: Sample extracts are cleaned up on a Florisil column for which column cutpoints are regularly determined.

Florisil Column Cleanup of Extract (Default Procedure): Quantitatively transfer the extract and hexane rinses (3 × 1 mL) to a Florisil column (8 g, 2.0% deactivated) prepared as described previously. Elute the column with 10% dichloromethane in hexane (approximately 35 mL) and collect the eluate in a round bottom flask or "Syncore Analyst" tube. Use the actual elution volume determined from the Florisil cutpoint determination of the particular batch of Florisil. The extract is ready for microvialling.

Additional Cleanup (Optional Procedure): The following cleanup procedure can be applied to samples which contain additional interferences that are not being removed using the standard chromatographic cleanup techniques.

Transfer the extract with hexane rinses (approximately 2 mL total volume) from the GC/MS microvial to a clean centrifuge tube. Add 1 mL 67%–70% sulfuric acid to the extract and vortex for 30 s. Allow the extract to stand until two distinct layers appear.

Remove the sulfuric acid (lower) layer from the centrifuge tube using a pipette. Repeat the previous procedure up to two times if the first sulfuric acid layer shows any yellow in appearance.

After the final acid wash, repeat the preceding steps with 2 mL of ultra-pure water instead of acid.

After the final water wash, add powdered anhydrous sodium sulphate (Na_2SO_4) to the extract and vortex for 30 s.

Prepare a glass wool column by placing a 10 mm plug of silanized glass wool into the end of a clean Pasteur pipette, and pre-elute with toluene and hexane. Transfer the extract to the column and elute the column with hexane rinses of the centrifuge tube (3 × 1 mL). Collect the extract in a clean centrifuge tube. The extract is ready for microvialling.

Preparation for GC/MS Analysis: After the final column step, concentrate the eluate to 1 mL by rotary evaporation or Syncore Analyst. Transfer the extract to centrifuge tube with 3× hexane rinses. Concentrate the extract to 300 μL under a gentle stream of nitrogen. Add nonane (30 μL) to a clean amber glass autosampler vial (the type used for high resolution MS analysis). Mark the vial at the 30 μL volume. Transfer the extract to the autosampler vial and concentrate to 150–200 μL under a gentle stream of nitrogen. Rinse the centrifuge tube with 350 μL of hexane and add the rinse to the autosampler vial. Concentrate the extract to a 150–200 μL using a gentle stream of nitrogen. Make sure the volume of extract does not go below 100 μL. Rinse the centrifuge tube with 350 μL of hexane and add the rinse to the autosampler vial. Concentrate the extract to 30 μL. Add an aliquot of recovery standard (10 μL) to the autosampler vial. Cap the autosampler vial. Store the vials in the freezer until just prior to GC/MS analysis.

Instrumental Analysis

Sensitivity: A sensitivity blank containing PCB 157 is analyzed with every batch. Sensitivity specifications are summarized in Table S3. If any of the instrumental QC criteria are not met, sample analysis cannot proceed.

Calibration: The default bracketing calibration procedure uses the mean RRFs from the analysis of the mid-level calibration solution run before and after the samples to calculate analyte concentrations. Initial calibration may be performed using a series of five solutions that encompass the working concentration range of the instrument. The calibration solutions contain a suite of labelled surrogates, recovery standards and target compounds.

Analysis: Analysis of the sample extract is performed on a low-resolution mass spectrometer (LRMS) equipped with a gas chromatograph (GC), and an auto-sampler, operating on manufacturer's software. AJ & W DB-5 chromatography column (60 m, 0.25 mm i.d., 0.1 µm film thickness), or equivalent column is coupled directly to the MS source. The MS is operated at a minimum unit mass resolution in the electron ionization (EI) mode using multiple ion detection (MID) acquiring two characteristic ions for each target analyte and surrogate standard. A splitless/split injection sequence is used. The ions acquired are listed in Table S4. A typical extract volume is 40 µL; if necessary, extracts are diluted to bring all target responses within the calibration range. However, the 40 µL extract volume is required to meet the specified reporting limits. Typical operating conditions for the GC/MS are presented below (Table S8).

Table S8. Temperature and Conditions of Gas Chromatography.

**GC Temperature Program**		**General GC Conditions**	
Initial Temp (°C)	60	Injector Temp (°C)	280
Hold time (min)	1	Injector	Splitless, 2 min
Rate (°C·min^{-1})	25	Carrier Gas	Helium
Temp (°C)	150	Maximum Temp (°C)	325
Hold time (min)	0		
Rate (C°·min^{-1})	12	MS Conditions	
Temp (°C)	300	Electron Energy (eV)	70
Hold time (min)	5.9	Mass Resolution	Unit

Qualitative and Quantitative Determination

A chromatographic peak is identified as a target compound if the following criteria are met for the quantification and confirmation ions:

1. Peak responses must be at least three times the background noise level;
2. The relative retention time must be within three seconds of the retention time predicted from the initial calibration runs and the surrogate standard retention times;
3. Peak maxima for quantification and confirmation ions must coincide within two seconds;
4. The relative ion abundance ratios must be within 20% of the theoretical.

Target concentrations are determined with respect to a labelled surrogate as shown in Table S9. Mean relative response factors (RRFs), determined from a mid-level calibration standard run at the beginning and end of the samples (called bracketing calibrations), are used to convert raw peak areas in sample chromatograms to final concentrations as follows:

$$\text{Concentration of Target} = \left(\frac{\text{area of Target}}{\text{area SUR}}\right) \times \left(\frac{\text{weight SUR (ng)}}{\text{RRF}}\right) \times \left(\frac{1}{\text{weight of sample (g or L)}}\right)$$

(ng/g or ng/L)

where $$\text{RRF} = \left(\frac{\text{area of Target}}{\text{area SUR}}\right) \times \left(\frac{\text{concentration SUR}}{\text{concentration of Target}}\right)$$ and SUR = the surrogate standard.

The PCBs are quantified against a labelled surrogate standard added at the beginning of the analysis procedure and are, thereby, recovery corrected. Surrogate recoveries are determined similarly against the recovery (internal) standard and are used as general indicators of overall analysis success.

Table S9. Analyte ions monitored, surrogates used and RRF determination for PCB congeners by GC/LRMS. (No entry in the "RRF Used" field designates an RRF derived from that same compound).

Analyte Name	Quantified against Labeled Standard	RRF Determination	Typical Retention Time	RT Win. (±s)	Mass1	Mass2	Theoretical m2/m1 Ratio (%)	Ion Ratio Tolerance (±%)
PCB 74	$^{13}C_{12}$-PCB 101		13.54	3	292	290	78.2	20
PCB 99	$^{13}C_{12}$-PCB 101		14.09	3	326	328	64.0	20
PCB 118	$^{13}C_{12}$-PCB 101		14.96	3	326	328	64.0	20
PCB 138	$^{13}C_{12}$-PCB 101		15.73	3	360	362	80.0	20
PCB 146	$^{13}C_{12}$-PCB 101		15.20	3	360	362	80.0	20
PCB 153	$^{13}C_{12}$-PCB 101		15.29	3	360	362	80.0	20
PCB 156	$^{13}C_{12}$-PCB 101		16.46	3	360	362	80.0	20
PCB 187	$^{13}C_{12}$-PCB 180		15.98	3	394	396	95.9	20
PCB 180	$^{13}C_{12}$-PCB 180		16.72	3	394	396	95.9	20
PCB 170	$^{13}C_{12}$-PCB 180		17.15	3	394	396	95.9	20
PCB 194	$^{13}C_{12}$-PCB 194		18.10	3	428	430	112.4	20
**Labelled Surrogates**								
$^{13}C_{12}$-PCB 101	$^{13}C_{12}$-PCB 153		14.01	3	338	340	64.0	20
$^{13}C_{12}$-PCB 180	$^{13}C_{12}$-PCB 153		16.72	3	406	408	95.9	20
$^{13}C_{12}$-PCB 194	$^{13}C_{12}$-PCB 153		18.10	3	440	442	112.4	20
**Recovery Standard**								
$^{13}C_{12}$-PCB 153	external		15.29	6	372	376	34.1	20

S3. Weight Loss and PCB Data from Non-Compliant Participants Following WM (Phase 2) (Table S10)

Table S10. Weight loss and PCB data from non-compliant participants following WM (Phase 2).

Variable	mP-CR (n = 6)	HH (n = 1)
Body weight		
Body Weight (kg)		
Baseline (week-13)	109.6 ± 24.6	86.3
Week-64	119.8 ± 24.9	99.6
PCB congeners (ng/g lipid)		
PCB 74		
Baseline (week-13)	6.7 ± 4.8	3.2
Week-64	5.6 ± 3.5	
PCB 99		
Baseline (week-13)	4.1 ± 1.8	4.7
Week-64	2.4 ± 1.9	
PCB 118		
Baseline (week-13)	12.4 ± 12.9	2.7
Week-64	5.6 ± 6.5	
PCB 138		
Baseline (week-13)	16.7 ± 15.3	11.5
Week-64	14.1 ± 12.9	
PCB 146		
Baseline (week-13)	2.4 ± 2.5	1.8
Week-64	3.2 ± 2.8	
PCB 153		
Baseline (week-13)	20.3 ± 18.3	13.2
Week-64	16.2 ± 15.2	
PCB 156		
Baseline (week-13)	3.1 ± 1.8	4.0
Week-64	3.2 ± 1.9	

PCB 170		
Baseline (week-13)	6.2 ± 4.2	12.8
Week-64	4.2 ± 2.6	
PCB 180		
Baseline (week-13)	18.1 ± 12.6	31.3
Week-64	11.0 ± 8.6	
PCB 187		
Baseline (week-13)	4.1 ± 3.2	4.0
Week-64	2.9 ± 2.2	
PCB 194		
Baseline (week-13)	3.7 ± 2.5	6.6
Week-64	2.2 ± 0.9	
Total PCB		
Baseline (week-13)	97.7 ± 72.2	95.8
Week-64	70.5 ± 58.1	

All values are expressed as mean ± SD.

© 2017 by the authors; licensee MDPI, Basel, Switzerland. This article is an open access article distributed under the terms and conditions of the Creative Commons by Attribution (CC-BY) license (http://creativecommons.org/licenses/by/4.0/).
